# Age-Related Shifts in Bacterial Diversity in a Reef Coral

**DOI:** 10.1371/journal.pone.0144902

**Published:** 2015-12-23

**Authors:** Alex D. Williams, Barbara E. Brown, Lalita Putchim, Michael J. Sweet

**Affiliations:** 1 Molecular Health and Disease Laboratory, Environmental Sustainability Research Centre, College of Life and Natural Sciences, University of Derby, Derby, United Kingdom; 2 School of Biology, Newcastle University, Newcastle upon Tyne, United Kingdom; 3 Environmental Research Institute, North Highland College, Caithness, United Kingdom; 4 Phuket Marine Biological Center, Phuket, 83000, Thailand; U.S. Geological Survey, UNITED STATES

## Abstract

This study investigated the relationship between microbial communities in differently sized colonies of the massive coral *Coelastrea aspera* at Phuket, Thailand where colony size could be used as a proxy for age. Results indicated significant differences between the bacterial diversity (ANOSIM, R = 0.76, p = 0.001) of differently sized colonies from the same intertidal reef habitat. Juvenile and small colonies (<6cm mean diam) harboured a lower bacterial richness than medium (~10cm mean diam) and large colonies (>28 cm mean diam). Bacterial diversity increased in a step-wise pattern from juveniles<small<medium colonies, which was then followed by a slight decrease in the two largest size classes. These changes appear to resemble a successional process which occurs over time, similar to that observed in the ageing human gut. Furthermore, the dominant bacterial ribotypes present in the tissues of medium and large sized colonies of *C*. *aspera*, (such as *Halomicronema*, an *Oscillospira* and an unidentified cyanobacterium) were also the dominant ribotypes found within the endolithic algal band of the coral skeleton; a result providing some support for the hypothesis that the endolithic algae of corals may directly influence the bacterial community present in coral tissues.

## Introduction

Different coral species have been shown to host distinct microbial communities, though these microbes are often similar between colonies of the same species despite separation of habitats by hundreds of miles [1]. There are likely to be many factors explaining why this may occur, ranging from the immediate environment to the physiology of a specific coral species. Indeed, one specific driving factor for these patterns may be the composition of the coral mucus [2–4]. The mucus of corals (and other organisms) is known to be a medium where the colonisation of certain bacterial species can occur along with the exclusion of others [2]. However, to date this has only been demonstrated under cultured conditions in controlled laboratory settings [2]. Surviving mucus-associated microbes have been shown to contribute a number of antimicrobial properties to their coral hosts, a finding which aids protection from potentially invasive and pathogenic bacteria [2,5]. This role is analogous to the function of commensal bacteria in many other organisms, for example in the mucus of the human gut, where shifts in this relatively stable community can result in shifts to a diseased state [6]. Parallels have already been drawn between the mucus of the human respiratory and gastro-intestinal tracts and mucus on the surface of corals [7,8], the coral gastrovascular cavity and also the coral lumen [9]. Such an analogy has been noted in a recent review on the coral holobiont [10].

Although there are many studies which have assessed the microbial communities associated with both healthy and diseased corals, little is known of the bacterial associates of the coral *Coelastrea aspera*, the subject of the present investigation. However, the environmental physiology of this species has been extensively studied [11–15]. Most recently, this physiologically robust species has shown an unexpected decline at sites around Phuket following a severe bleaching event observed in 2010 [16]. One proposed explanation for this demise, was that of senescence [16]. Interestingly, colonial animals such as corals have historically and theoretically been described as non-ageing organisms [17]. However, recent studies on certain species of reef coral appear to show declines in physiological performance with age [18–20]. One aspect which has been well documented to be strongly dependent on age in numerous higher organisms, is the relationship hosts share with their associated ‘natural’ microbiota throughout their life cycle [21,22]. For example, microbial communities associated with the avian cloaca have been shown to significantly alter with the age of the host [22]. Furthermore, humans often exhibit marked shifts in their microbial communities, a result again often associated with aging. Interestingly this has been documented to occur both externally [23] and internally (i.e. within the human gut) [21,24,25]. We therefore considered that investigation into the microbial communities of different sizes of corals (using size as a proxy for age), might provide some insight into whether corals show similar age-related patterns as those described in higher organisms. In this preliminary study we examined the diversity of microbial communities associated with different size-classes of the reef coral *C*. *aspera*, at a site on Phuket Island, Thailand; where the size and age structure of this particular species are well known [16].

## Methods

### Field Permit

Field permits were obtained from the National Research Council of Thailand (foreign researcher ref no. 208807221 project no. 002/5457).

### Field Sampling

To compare the microbial communities of the merulinid, *Coelastrea aspera*, different size classes of the coral were identified on an intertidal reef flat on the south-east coast of Ko Phuket, Thailand (7°50′ N 98°25.5′E) during March 2014. All samples were collected on one date at the end of a neap tide sequence from an area of intertidal reef flat measuring ~20m x 50m.

Five size classes of colonies were sampled based on measurement of mean diameters calculated from the maximum colony diameter and corresponding diameter at right angles to this dimension. The mean diameter and standard deviation for each colony size class were as follows; juveniles—mean diam 2.8±0.96cm; small—mean diam 6 ±0.83cm; medium—mean diam 9.9±2.10cm; large microatoll (with ~30–40% mortality)—mean diam 28.7±3.92cm and larger entire colonies—mean diam 32.16±3.20cm) (S1 Fig). The estimated age of these corals was based on earlier alizarin staining [26], and corresponds to juvenile samples being ~1y; small ~2-3y; medium ~4y; large microatolls ~9-10y; and larger entire colonies ~10-12y (S1 Fig). Although colonies selected for sampling were all located within a very restricted area of the reef, there were subtle differences in their tidal environment. Juvenile and large entire colonies were sampled from a shallow channel where water receded, on the largest spring tide, approximately 30 minutes later than that of the slightly higher reef flat table dominated by small, medium and large micro-atoll colonies.

Samples for microbial analysis were taken using 1.5cm diam hole-punches (which were sterilized between each individual sample) from the east side of colonies in each of the five size classes. A single plug sample was extracted from each of the 5 colonies within a size class. Samples were obtained from half way down the colony from a point midway between the base of the colony and its apex.

Since *C*. *aspera* is capable of both fission and fusion, which could confound any size/age relationship [17], great care was taken in selection of coral colonies for sampling. Every effort was made to select colonies which were unambiguous in terms of their growth form i.e. they were isolated from other colonies by dead reef substrate and were perfectly spherical in shape in the case of juvenile-medium sized colonies. Our prior knowledge of this population of *C*. *aspera*, whose demography we have studied over the last 36y from the settling of recruits in the early 1990s through their development to the largest colonies [16], assisted in this task.

In addition to assessing size-related shifts in microbial communities we also aimed to assess how the microbial communities of the different coral compartments (mucus, tissue, skeleton and endolithic algae) varied in these corals. For this, we sampled a further set of medium sized corals (mean diam 9.9±2.10cm). Each of the above compartments was initially sampled from 5 independent medium-sized coral colonies. However upon extraction only 3 samples were able to be used for the mucus samples and 4 for the skeleton. The full complement (n = 5) was extracted for both the tissue and the endolithic algae. Coral plugs were collected (as in the initial protocol) and stored in separate sterile containers. These were then transported back to the laboratory for extraction of microbial communities from specific compartments. The cores were first centrifuged in 50 ml falcon tubes to collect the surface mucus layer. The tissue was then air brushed off into sterile zip lock bags. The resulting skeleton was divided into skeleton with no visible endolithic algae and skeleton with endolithic algae. All four compartments, for each of the five colonies, were then stored in 100% ethanol and treated as described below. The different compartments described in this study are shown in S2 Fig, with plugs being collected midway between base and apex of each medium-sized colony.

### 16S rRNA Gene Bacterial Diversity

DNA for 16S rRNA gene bacterial analysis was extracted from these samples using the QIAGEN DNeasy Blood and Tissue kit following the protocols outlined in [27]. 30 μl PCR reactions were carried out for each sample in a Hybaid PCR Express thermo cycler, each consisting of 1.5 mM of MgCl_2_, 0.2 mM of dNTP (PROMEGA), 0.5μM of universal bacterial primer 357f (5’-CAGCACGGGGGGCCTACGGGAGG CAGCAG-3’) and 518r (5’-ATTACCGCGGCTGCTGG-3’) [28], 2.5 μl of Taq DNA polymerase (QBiogene), and 20 ng of template DNA. PCR protocols consisted of thirty PCR cycles at 94°C for 30 sec, 53°C for 30 sec and 72°C for 1 min, with a final extension at 72°C for 10 min [28]. Two types of non-culture molecular techniques were utilized in this study, DGGE and 454 pyrosequencing. PCRs for DGGE were conducted with the addition of a GC rich clamp on the forward primer [29] and for 454 sequencing, HotStarTaq polymerase (Qiagen) was substituted to the PCR mix to provide more specificity. Representative bands were selected from the DGGE profiles and sequenced following the protocol outlined in [27]. For 454 analysis, the PCR products were cleaned using AMPure magnetic beads, quantified using the Qubit flourometer (Invitrogen) and pooled to an equimolar concentration. Sequences were then run on 1/8^th^ of a 454 FLX Titanium pico-titer plate at Newgene in the Centre for Life, Newcastle, UK. Technical control samples were processed in the same way as above, whereby samples consisting of just ethanol (used for preservation of the samples) were extracted and sequenced as above. No DNA was detectable either after PCR or from the downstream processing.

Pyrosequences from the 454 analysis were processed using the QIIME pipeline (version 1.5.0) [30], using the SILVA reference database (release version SSU/LSU 123) [31]. Sequences were filtered as referenced in [32]. In brief, this consisted of discarding sequences if they were found to fit into any of the following criteria; (i) < 50 nucleotides, (ii) contained ambiguous bases (Ns), and/or (iii) contained primer mismatches. Analysis using 2% single-linkage pre-clustering (SLP) and average-linkage clustering based on Pairwise alignments (PW-AL) was performed to remove sequencing based errors. The remaining sequences were denoised within QIIME. The resulting reads were checked for chimeras and clustered into 98% similarity operational taxonomic units (OTU) using the closed USEARCH algorithm in QIIME [33]. All singletons (reads found only once in the whole data set) were excluded from further analyses. After blast searches on GenBank, the best BLAST outputs were retained, i.e. the most complete identifications, and compiled on an OTU table, including all identified OTUs and respective read abundances. A total of 644,754 raw nucleotide reads were produced with an average length of 52 bp, corresponding to 198 Mb. After filtering, a total of 20,950 quality reads were acquired. The length of the remaining sequences varied from 95 bp to 151 bp, with an average length of 126 bp.

For DGGE, gels were digitally converted and processed in Phoretrix 1D Pro, producing a band matrix with operational taxonomic units expressed in both presence-absence and relative density. Analysis was conducted using PRIMER 6.0 [34] for both sets of data. Results highlighted the same trends regardless of technique, despite 454 analysis being considered as offering a more comprehensive analysis of community structure [35]. A one-way Analysis of Similarities (ANOSIM) indicated the extent that variations between microbial populations could be attributed to sample class (size/age). SIMPER analysis of Bray-Curtis similarity was employed to assess the dynamics of specific microbe relative abundances between the size/age categories. Finally, a non-metric multidimensional scaling (NMDS) plot was produced based on the Bray-Curtis similarity analysis.

### Microscopy

Samples for scanning electron microscopy (SEM) (n = 5 of medium-sized colonies only) and immuno-histological analysis (n = 5 of medium-sized colonies) were preserved in 50 ml falcon tubes containing 5% paraformaldehyde for 24 hr and subsequently stored in 100% EtOH [36].

### Scanning Electron Microscopy (SEM)

Samples for SEM were dehydrated using 25, 50 and 75% ethanol (30 mins each), followed by a period (2 x 1 hr) in 100% ethanol, with final dehydration consisting of air drying for 1 hr. Specimens were then mounted on an aluminium stub with Achesons Silver Dag (dried overnight) and coated with gold (standard 15nm) using an Emi Tech K550X Splatter Coating Unit. Specimens were examined using a Stereoscan 240 scanning electron microscope, and digital images collected by Orion 6.60.6 software.

### Immuno-Histology

Samples for immuno-histology were decalcified and embedded in paraffin wax. All tissue sections were cut to a thickness of 1 μm. Survey sections were stained using the general DNA stain toluidine blue [37]. The fluorescent stain acridine orange was applied to tissue sections [38] for detection of bacterial cells. Acridine orange was applied to tissues by pipetting the stain directly over cut sections which were left for 10 min. Excess stain was removed by gently washing with distilled water. Sections were viewed at magnification x 400 using a Leica DMRB light microscope and images obtained with an integrating camera (QICAM Fast 1394).

## Results

### 454 Sequence Data and Read Number

After initial filtration of the 454 sequence data (see methodology) a total of 20,950 high-quality pyrotag sequencing reads were obtained from the sample set (total *n* = 25 individual coral samples from all size classes). This result is comparable to other studies which have used similar techniques [32,39]. The samples contained between 300 and 1,350 reads (∼838 reads/sample), with average sequence lengths ranging from 95 to 151 bp per sample after primer and bar code removal. However, after the removal of all singletons (reads found only once in the whole data set—see methods) and after blast searches on GenBank, only relatively few unique sequences were retained (n = 26 unique reads). These sequences were submitted to GenBank under the unique accession numbers KT962875-KT962900. Although a relatively low bacterial richness was observed with all the samples, we believe this result is unlikely to be a processing error as control samples (those consisting of the ethanol used in the preservation) showed no PCR product and no results were obtained from the further downstream processing of these samples. We have discussed likely reasons for this lower than expected bacterial richness in the discussion.

### Size/Age Related Bacterial Assemblages in the Coral *C*. *aspera*

**Fig 1:**
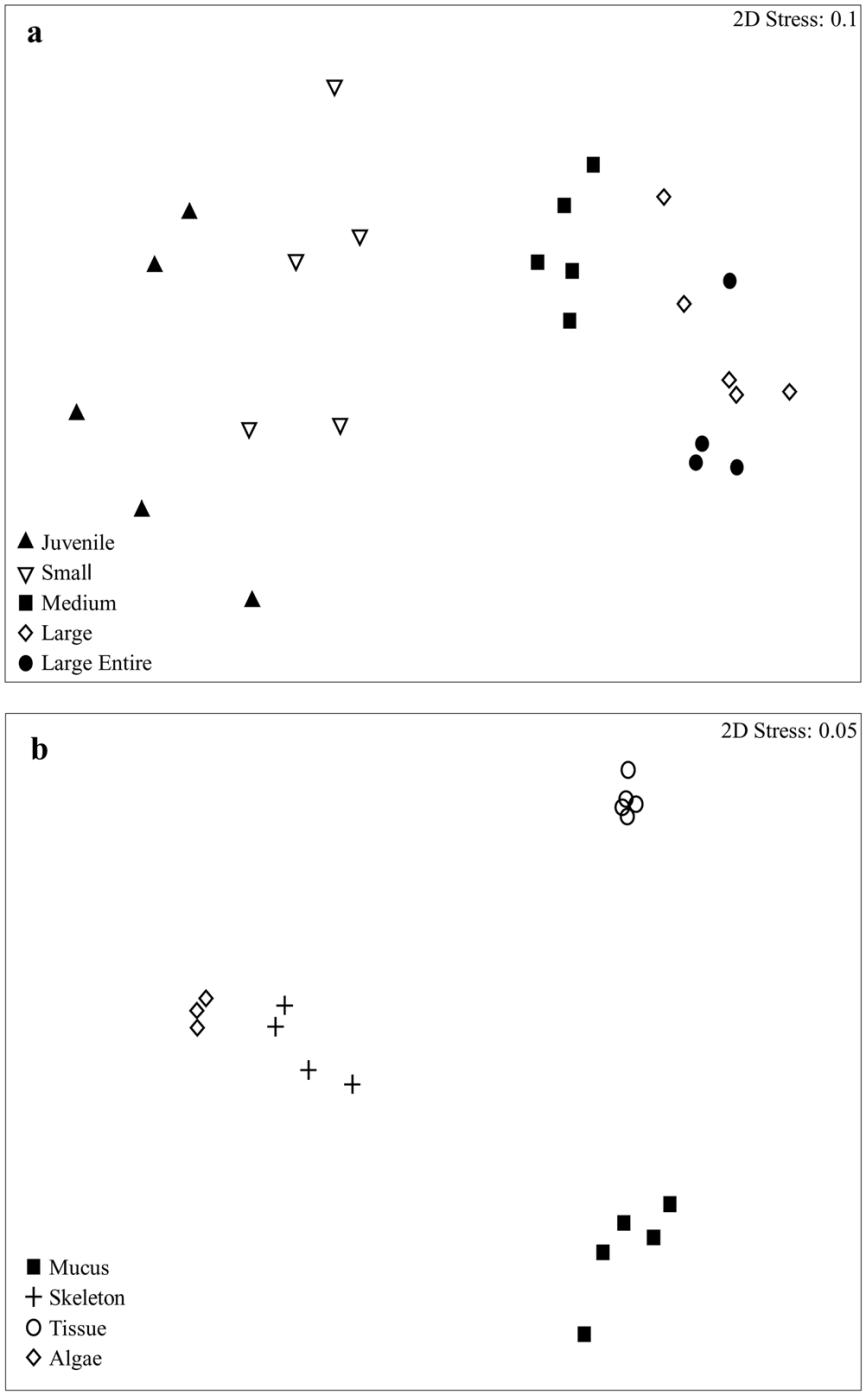
NMDS showing variation and similarities in 16S rRNA gene bacterial community structure associated with different samples. (a) variation in bacterial community structure between juvenile, small, medium, large microatolls and large entire colonies of *Coelastrea aspera*. (b) variation in bacterial community structure between different micro-compartments associated with a coral core of a medium sized colony of *C*. *aspera*.

**Fig 2:**
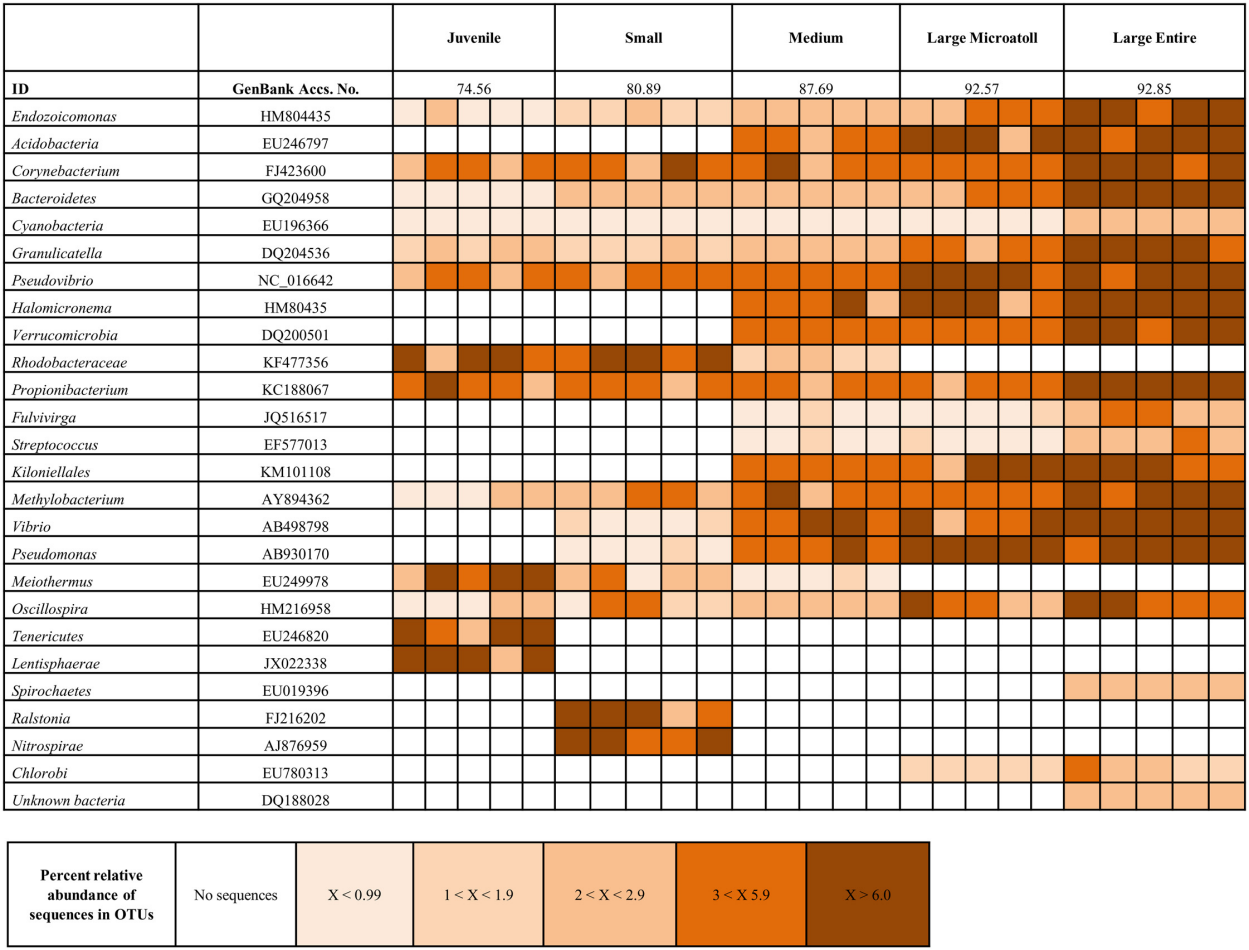
Heatmap, illustrating the dominance of different bacteria present in various samples of *Coelastrea aspera*. Columns correspond to individual coral replicates. Colours represent the relative abundance of bacterial ribotypes recovered across all samples; the darker the colour the more abundant the bacterium present within the sample type. See key for OTU density number from 454 analysis. Second row displays SIMPER analysis, highlighting percent similarity of all replicates within each sample type. The table also shows the closest match of the sequence on GenBank and associated Accession Number.

There was a significant difference in the 16S rRNA gene bacterial diversity between all size classes of *C*.*aspera* sampled in this study. Interestingly, similar differences in the bacterial diversity were found to occur irrespective of the profiling methodology i.e. DGGE and 454 analysis (ANOSIM, R = 0.529, p = 0.001; R = 0.76, p = 0.001 respectively). As a result, we only report the results for the latter in the following section. Pair-wise post hoc tests showed that the observed differences (Fig 1a for 454 sequence analysis NMDS) occurred between all size classes (R< 0.97, p < 0.04). Larger corals (those classed in the groups: larger entire colonies, large microatolls and medium) showed a greater bacterial richness than small corals and juveniles (Fig 2). Juvenile corals were characterised by a Shannon Diversity index value (H^1^) of 2.84 ± 0.28, with small, medium, large microatolls and larger entire corals exhibiting 2.94 ± 0.27, 3.14 ± 0.08, 3.09 ± 0.14 and 3.03± 0.14 respectively. Note the slight decrease in diversity in the two larger size classes. There was also greater variation in bacterial diversity associated with replicates of the smaller colonies (SIMPER analysis—80% similarity between replicates) and juveniles (74.56%) compared to the variation seen in medium (87.69%), large microatolls (92.57%) and large entire colonies (92.85%).

Juvenile corals were dominated by ribotypes related to *Corynebacterium*, *Pseudovibro*, *Rhodobacteraceae*, *Propionibacterium*, *Meiothermus*, *Tenericutes* and *Lentisphaerae*. Small colonies showed a similar microbial community to that of the juveniles (Fig 2), however two bacterial sequences closely resembling a *Ralstonia* sp. and a *Nitrspirae* sp. which were dominant in the small-size colonies were absent in all the juvenile samples (Fig 2). These two bacteria (the *Ralstonia* sp. and the *Nitrspirae* sp.) were also not detectable in the larger colonies (medium, large microatoll and large entire colonies) (Fig 2). These ‘older’ colonies were dominated by a different group of bacteria (Fig 2) which included; *Vibrio*, *Streptococcus*, *Pseudomonas*, *Kiloniellales*, *Halomicronema*, *Acidobacteria*, and *Verrucomicrobia*, the latter four of which were undetectable in ‘younger’ corals.

### Endolithic Algae as Contributors to the Tissue Bacterial Assemblage

**Fig 3:**
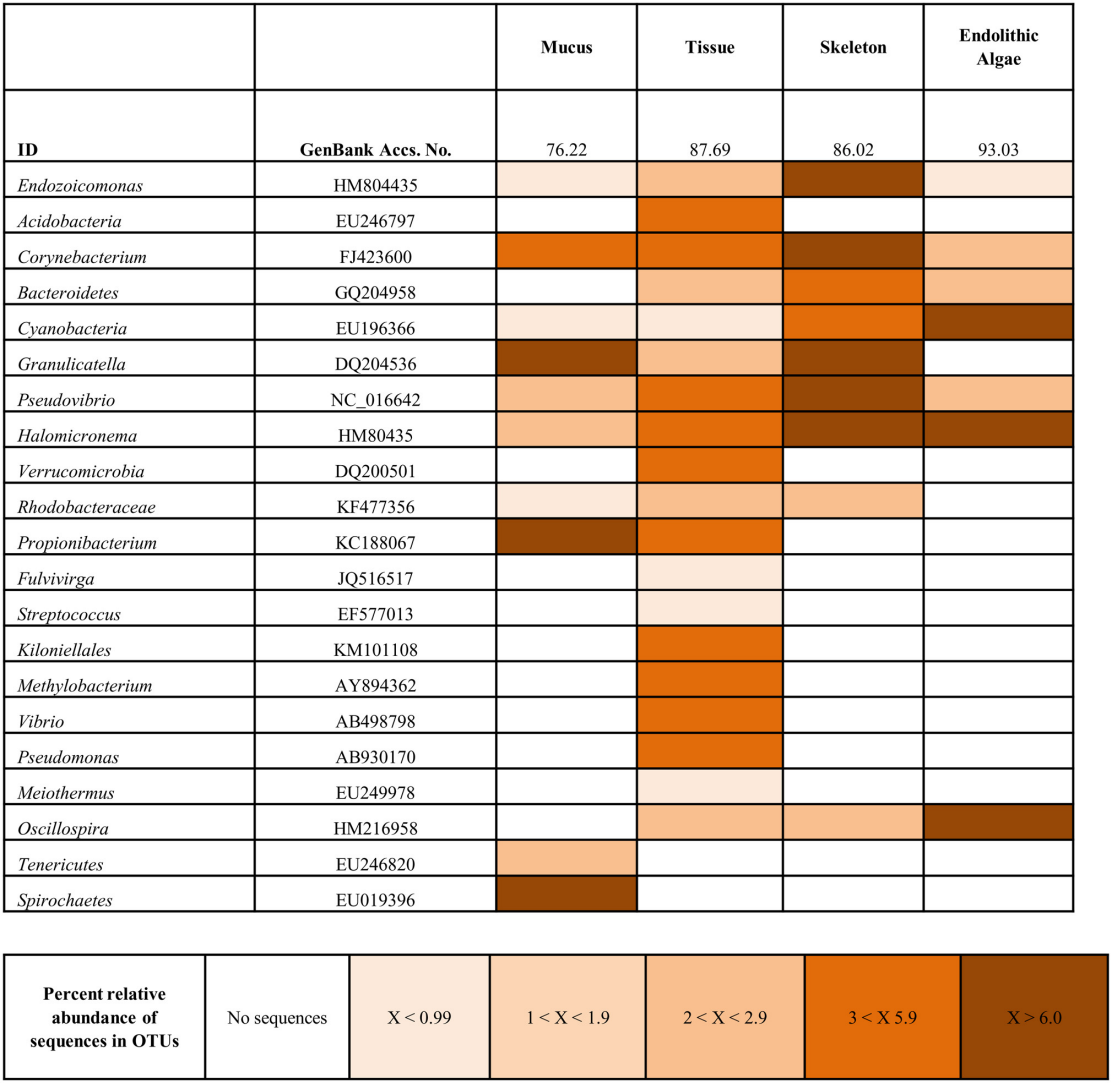
Heatmap, illustrating the average dominance of bacteria present in the compartmentalised samples of *Coelastrea aspera*. Colours represent the averaged relative abundance of all replicates within each sample type. The darker the colour the more abundant the bacterium present within the sample type. See key for OTU density number from 454 analysis. SIMPER analysis values shown in the second row. The table also shows the closest match of the sequence on GenBank and associated Accession Number.

There were significant differences (454 analysis—ANOSIM, R = 0.96, p = 0.001) between the 16S rRNA gene bacterial diversity associated with the different tissue compartments (mucus, tissue, skeleton and endolithic algae). Variation between the community structure of these compartments is shown in Fig 1b. The tissue samples, (which aimed to only sample the tissue but may have included parts of the skeleton and mucus) showed the closest similarity between replicates in the main part of the analysis (see Fig 1a and 1b). The mucus samples showed two ribotypes (a *Tenericutes* and a ribotype related to a *Spirochaetes*) which appeared absent in the tissues of the same sized colony (Fig 3). Interestingly, while *Tenericutes* was only detected in the smaller (younger) colonies, the *Spirochaetes* ribotype was detected only in the larger (older) colonies (Fig 2). Skeletal samples showed a lower bacterial species richness to those found in the tissues (19 ribotypes in the tissue, compared to 9 in the skeleton), however they did share some similar ribotypes (Fig 3). These included the *Endozoicomonas*, the *Corynebaterium*, the *Bacteroidetes*, the *Granulicatella*, the *Pseudovibrio*, the *Halomicronema*, the *Rhodobacteraceae*, and the *Oscillospira* (Fig 3). In addition, the endolithic algal band also shared certain bacterial ribotypes with those found in both the tissues and the skeleton (Fig 3). Specifically the algal band was dominated by six ribotypes including *Corynebacterium*, *Bacteroidetes*, a *Cyanobacterium*, *Pseudovibrio*, *Halomicronema* and *Oscillospira* (Fig 3). However, it must be noted that the endolithic algal band would also include both eukaryotic and phototrophic organisms, which we did not assess in this study.

**Fig 4:**
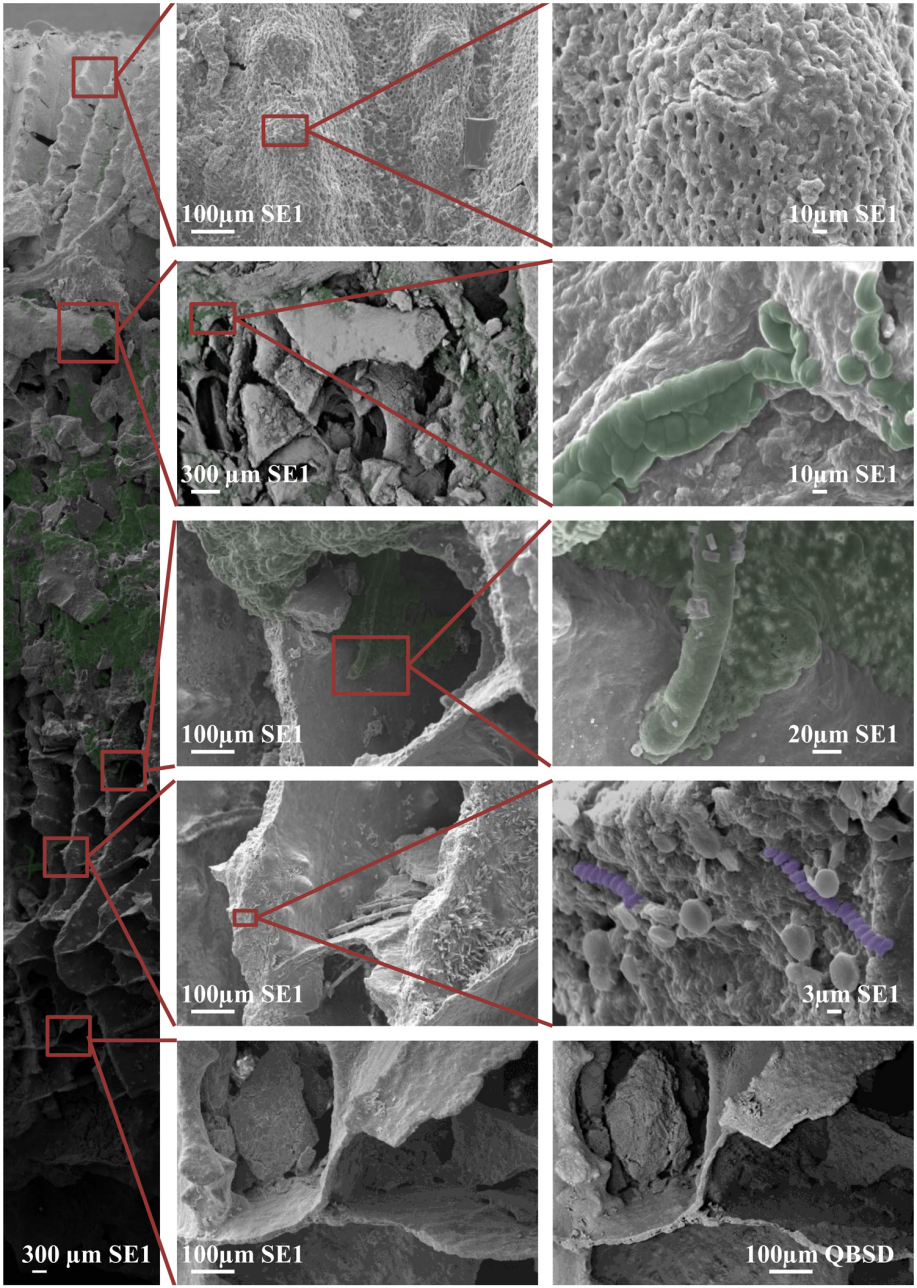
Scanning electron micrographs of preserved coral core of *Coelastrea aspera*. SEM highlighting the presence and absence of numerous microorganisms including the cyanobacterium *Halomicronema* sp. (green) and cocci (purple). Left hand image represents a longitudinal section through a core, with former living surface uppermost and skeleton at base, while other images are magnified areas of regions in the LS depicted by boxes and red guide lines. Individual scale bars shown on each image. SE1 = Secondary Electron, QBSD = Quadrant Back-Scattering Detector.

**Fig 5:**
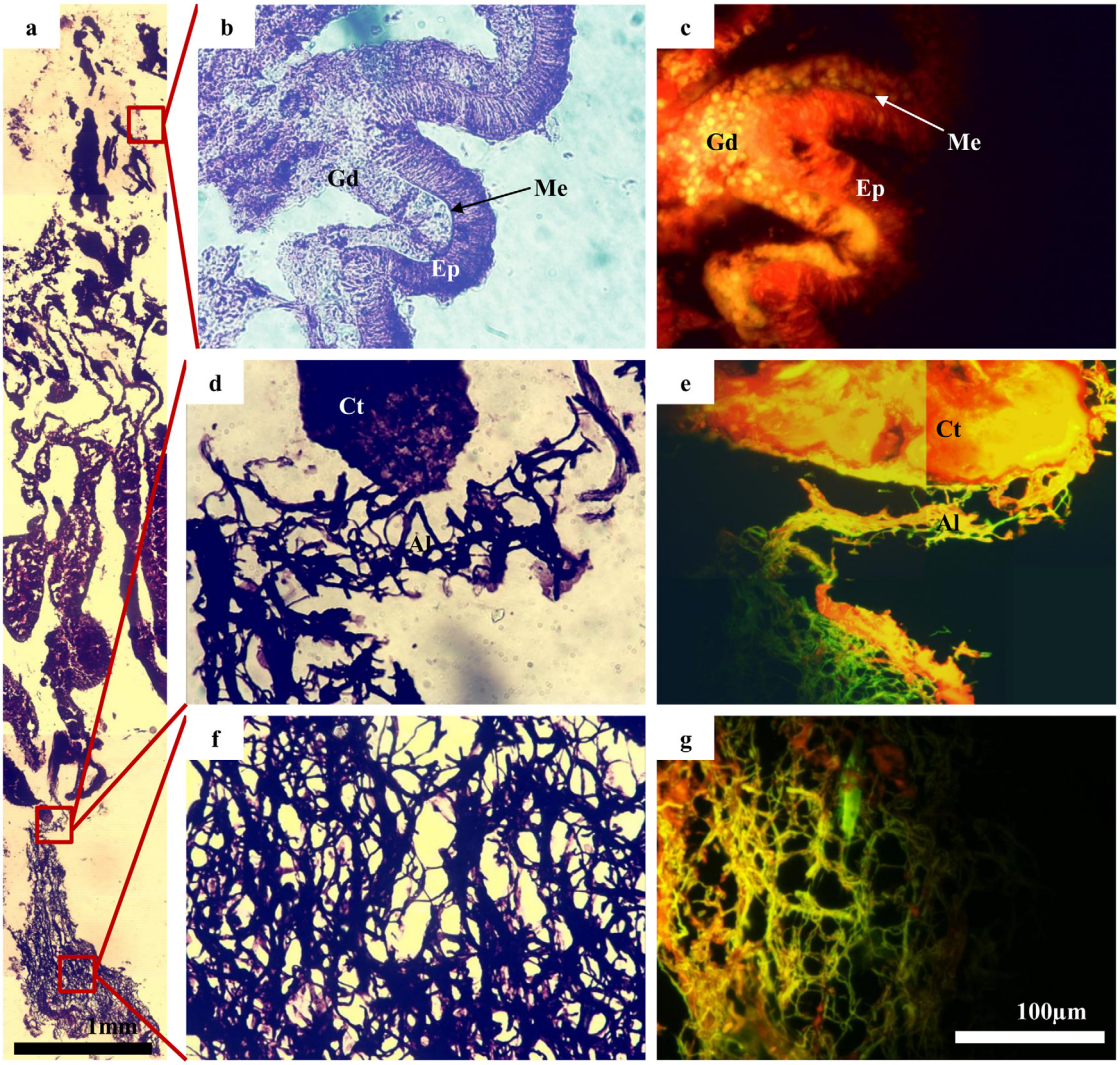
Immuno-histological sections of the coral *Coelastrea aspera*. Images highlight, in particular, the close association between the coral tissue and the cyanobacterium *Halomicronema* sp. known to dominate the endolithic algal band in many Indo-Pacific coral species. Panel (a) represents a longitudinal section (LS) of the coral with the oral surface uppermost and the endolithic algal layer at the base. Panels (b)—(g) show magnified and stained areas in LS. Panels (a), (b), (d), (f) stained toluodine blue (acidic structure affinity); (c), (e), (g) stained acridine orange (yellow fluorescence = dsDNA, Red/orange fluorescence = ssDNA and RNA). Gd = gastrodermis; Me = mesoglea; Ep = epithelium; Ct = coral tissue; Al = algal tissue. Panels (b)—(g) at same magnification. Scale bar 100 μm.

The SEM imagery showed the presence of at least two distinct cocci bacteria within the skeleton (for a representative image see Fig 4). These same bacteria were detected in all replicate samples assessed. Using 16S rRNA bacterial analysis (see above), these bacteria may be the *Bacteroidetes* and the *Pseudovibrio*. However, it remains impossible to confirm this with the methodology employed here. In the endolithic algal band, at least two types of cyanobacteria were also identified through SEM imaging (Fig 4). Based on the sequencing, these may be a combination of the *Halomicronema*, and/or the unidentified cyanobacterium. At least one of these cyanobacteria showed penetration from the skeleton into the coral tissues (Figs 4 and 5). This suggests a possible direct link between the endolithic algal band and the coral tissue itself. Notably, although sequencing revealed a relatively large number of bacteria associated with the healthy tissue and the mucus; no bacteria were observed to be associated with the surface of the coral tissue during imaging.

## Discussion

The significant differences observed here between the bacterial diversity occurring in various size classes of *C*. *aspera*, potentially indicates age-related shifts in the coral’s natural ‘healthy’ microbial associates. The lowest bacterial species richness was associated with juvenile and small colonies (<6m mean diam) and the highest richness in medium (~10cm mean diam) and larger colonies (>28cm mean diam).

Interestingly, although we use the term highest bacterial richness above, this is relative, as the overall richness detected in this study was notably lower than those reported in other studies using similar techniques [32,36,39,40]. This is despite the fact that the samples here were processed and sequenced in exactly the same way as other studies by the same authors [32,36]. Therefore, a more likely explanation for this observed lower richness may be that corals in more extreme environments, such as *C*. *aspera* living at the top of the intertidal zone, actually harbor fewer microbes than those in sub-tidal habitats. In support of this, studies which have assessed the microbial communities inhabiting extreme environments have reported similar reduced diversity [41,42,43]. Although we feel this is worthy of note, further research is needed to fully understand the implications of diversity and abundance of microbial associates of corals with relation to health status and tolerance to stress events. Sampling the same coral species and the same colony size from the same location across a gradient from exposed inter-tidal corals to those completely submerged would allow this hypothesis to be tested.

Regardless of the lower than expected richness, the observable differences in the coral microbial communities, noted between the different size classes, could be attributed to at least two factors; that of the environment and that of the physiology of the coral. Regarding the former, it is interesting to note that the microbial patterns observed do not conform to different aerial exposure times (i.e. juveniles and large entire colonies living under marginally but significantly less stress, in a shallow channel, compared with other sized colonies), but rather point to a relationship with the age/size of colonies. Furthermore, the observed differences and similarities in the bacterial communities do not appear to conform to the relative position that the samples were taken from within the water column. Samples from medium and large microatoll colonies, for example, were obtained from similar heights above the reef substrate, yet the microbial communities of these two size-classes of colonies are significantly different.

From the coral organism perspective a pattern of increasing microbial richness as the coral ages is perhaps not unexpected, since developmental changes which occur over the coral’s life-history likely result in more complex and varied niches being developed, which in turn would support the colonization of a more diverse prokaryotic assemblage. In this instance, certain bacteria such as *Corynebacterium*, *Granulicatella*, *Pseudovibrio*, and the *Propionibacterium* appear to remain throughout the growth of the coral, while others appear to be outcompeted (such as the *Meiothermus*, the *Tenericutes* and the *Lentisphaerae*). These latter bacteria are replaced with a larger number of different bacteria, presumably until an environmentally-dependent stable community is reached, reflecting that of a climax community [44]. The presence of certain bacteria, detectable within the smaller colonies and juveniles, yet undetected in medium and larger colonies, suggests a significant change in bacteria may be occurring during coral development. Specifically, species such as *Tenericutes*, *Lentisphaerae*, *Ralstonia* and *Nitrospirae* were only detected in juveniles and small-sized colonies. Furthermore, *Rhodobacteraceae* appeared to dominate juvenile and small colonies, occurring to a lesser extent in medium sized corals and undetectable in large microatoll and large entire colonies. Such a result supports recent evidence which shows that some corals acquire members of the Roseobacter clade-affiliated (RCA) group, (in which *Rhodobacteraceae* belong), early on in their life cycle [45,46]. However, it is still unclear how the early acquisition of such bacteria influences the coral host. It is noteworthy that RCA bacteria, have been shown, in some instances, to be both beneficial and detrimental to coral hosts. Some are known to exhibit antibacterial activity against certain marine pathogens [2,47,48] while others from this group have been described as possible pathogens [49,50]. Other members of this group have also been proposed as symbionts of the coral’s own symbiotic algae [51]. The importance of this latter relationship between bacterium and *Symbiodinium* remains largely unknown, however it has been suggested that the relationship may result in increased fitness to either the *Symbiodinium* and/or to the coral host directly [45,52]. More recently, RCA bacteria have also been implicated in aspects of coral development [45,52], with certain species being shown to proliferate in changeable environments [53], which could include habitats such as the intertidal reefs in the present study. Although the presence of these bacteria in the younger age classes is an interesting finding, we cannot, however, infer from these results the roles that any of these bacteria may play in juvenile and small colonies. Regardless, it remains clear that the patterns observed here, regarding shifts in the bacterial diversity between different size classes and similarities within, strongly suggests age plays a role in structuring these communities, at least in *C*. *aspera*.

One specific physiological factor potentially governing this observed succession of healthy microbial associates, may be the composition of the coral mucus and the rest of the already established bacterial community [45,52,54]. Interestingly, if age related shifts occur for all colonies of *C*. *aspera*, this species of coral may indeed show senescence. The increase of potentially pathogenic bacteria in the older class sizes, which are absent or rare in the younger corals, is one result which supports this theory. Such a finding could be viewed in two contrasting ways. Firstly, the older corals may be showing a lack of immune-competence linked with age, or alternatively, these older corals have had longer to acquire microbes from the surrounding environment. At the time of sampling none of the corals showed any signs of disease or stress, therefore we cannot draw a firm conclusion on the significance of this finding. Further work into the immune response of corals of different sizes may highlight if either of these scenarios is more likely. Should a reduction in immuno-competence be shown to be a factor then it would reflect similar trends as those described in the age-related shifts in microbial communities associated with higher organisms such as humans and birds for example [22,23,55,56].

It is also worth considering what factors distinguish the smaller colonies (<6m mean diam) from colonies larger than 10cm diameter (classed in this study as medium, large microatolls and larger entire colonies). Interestingly, in this particular coral species, at the given study site, a number of factors begin to affect colonies of 10cm diameter and above. These include the corals becoming fully reproductive [57–59], the potential of switching investment in tissue growth to that of skeletal growth [60] and increased vulnerability to solar stresses and partial mortality [11]. All these factors have implications for the energy balance of the coral, in particular the costs of reproduction [61,62] and environmental defenses [63]. These in turn may have an impact on mucus production which involves a major loss of dissolved organic carbon [64], particularly in *C*. *aspera* living in an intertidal environment where mucus is an essential protection against desiccation stresses [7]. It is therefore quite possible, though yet untested, that these major changes in the coral life-history might significantly affect both the quantity and content of the mucus layer and, as a result, the composition of the microbial community that it contains [10,65].

An interesting aside from the main study was the finding that some of the dominant bacterial ribotypes, associated with the tissues of medium-large sized *C*. *aspera* (such as the *Halomicronema*, the *Oscillospira* and an unidentified cyanobacterium), were also the most abundant in the endolithic algal samples, present in a distinct band within the skeleton. This result suggests that there may be some bacterial interaction between different compartments of the coral (skeleton, endolithic algal band, coral tissue and the surface mucus layer). Scanning electron micrographs and immuno-histology highlighted direct contact between the algal band and coral tissues with cyanobacterium species aggregated in recesses within the skeleton and, in certain instances, filaments projecting directly out of the coral skeleton (Figs 4 and 5). This is redolent of the boring cyanobacterium identified as a potential causal agent of black band disease [54]. Previous studies have proposed that close algal/tissue interactions may provide routes for microbial introduction [54, 66–68], and the findings shown here support this view. Such a direct linkage may lead to a greater understanding of how the endolithic algae are able to provide photo-assimilates which are translocated to the coral host during extreme bleaching events [69], a process believed to aid host survivorship and overall fitness during periods of elevated sea temperature.

Finally, we must address the issue of potential reagent contamination which has been observed in numerous next generation analyses of environmental samples. In particular, two specific ribotypes detected in this study, *Ralstonia* and *Propionibacterium* have been identified as possible contaminants in previous studies [70, 71]. However, these same genera have also been implicated as core members of the coral microbiome in a recent study [72]. As a result, we decided to include these ribotypes in our analysis. However, further study should be conducted with this aspect in mind, in order to assess whether techniques such as 454 pyrosequencing consistently misidentify specific community members and whether a standardized approach should be designed for future studies employing these methodologies.

## Conclusions

In conclusion, we have shown that bacterial assemblages vary significantly between different coral colony sizes yet remain relatively consistent between replicates of the same size class. The step-wise increase in bacterial diversity, with increasing size and age of colony (from juvenile to medium sized colonies), is noteworthy, and while it is impossible to say whether these changes confer advantages or disadvantages to the growing coral they do provide evidence of age-related processes. Ultimately only further research, following individual corals over a number of years, in a multidisciplinary study, will clarify the significance of these observed shifts.

## Supporting Information

S1 Fig Representative images of the five size categories of *Coelastrea aspera*. The mean diameter and standard deviation for each size class were as follows; juveniles—mean diam 2.8±0.96cm; small—mean diam 6 ±0.83cm; medium—mean diam 9.9±2.10cm; large microatoll (with ~30–40% mortality)—mean diam 28.7±3.92cm and larger entire colonies—mean diam 32.16±3.20cm. Estimates of age of these corals based on earlier alizarin staining (Hawkridge 1998) mean the juvenile samples are ~1y; small ~2-3y; medium~4y; large microatolls ~9-10y; and larger entire colonies ~10-12y.
(TIF)

S2 Fig Cross section of colony (diam ~7cm) highlighting the different microbial compartments in the coral *Coelastrea aspera*. SKL = skeleton; EA = endolithic algae; M = mucus and T = tissue.
(TIF)
