# Supplementary figures and images for: Age-Related Shifts in Bacterial Diversity in a Reef Coral
Source: PLoS One. 2015 Dec 23;10(12):e0144902. doi: 10.1371/journal.pone.0144902 (PMC4689413; doi:10.1371/journal.pone.0144902)

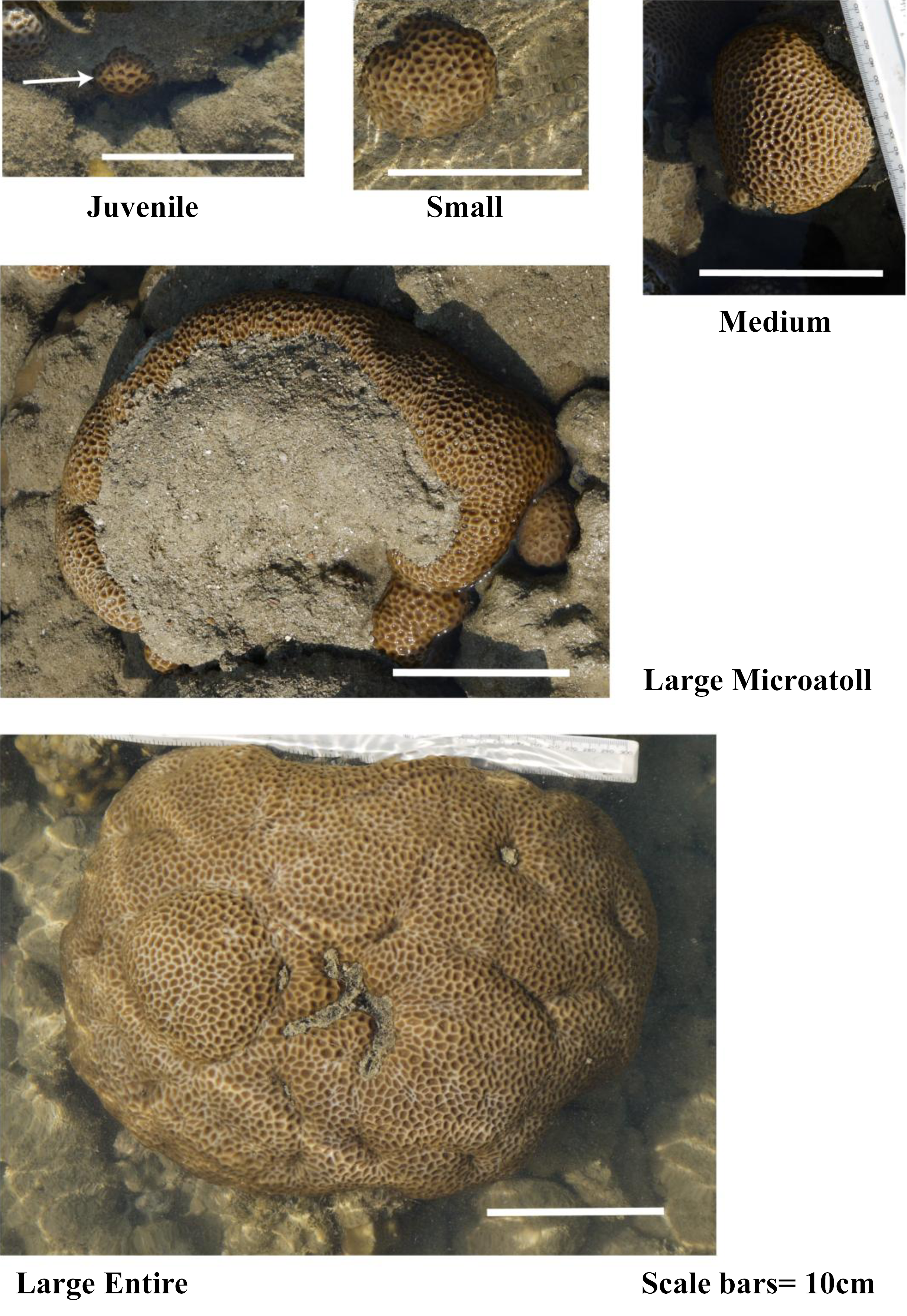

Supplement: S1 Fig — The mean diameter and standard deviation for each size class were as follows; juveniles—mean diam 2.8±0.96cm; small—mean diam 6 ±0.83cm; medium—mean diam 9.9±2.10cm; large microatoll (with ~30–40% mortality)—mean diam 28.7±3.92cm and larger entire colonies—mean diam 32.16±3.20cm. Estimates of age of these corals based on earlier alizarin staining (Hawkridge 1998) mean the juvenile samples are ~1y; small ~2-3y; medium~4y; large microatolls ~9-10y; and larger entire colonies ~10-12y. (TIF) [file pone.0144902.s001.tif]

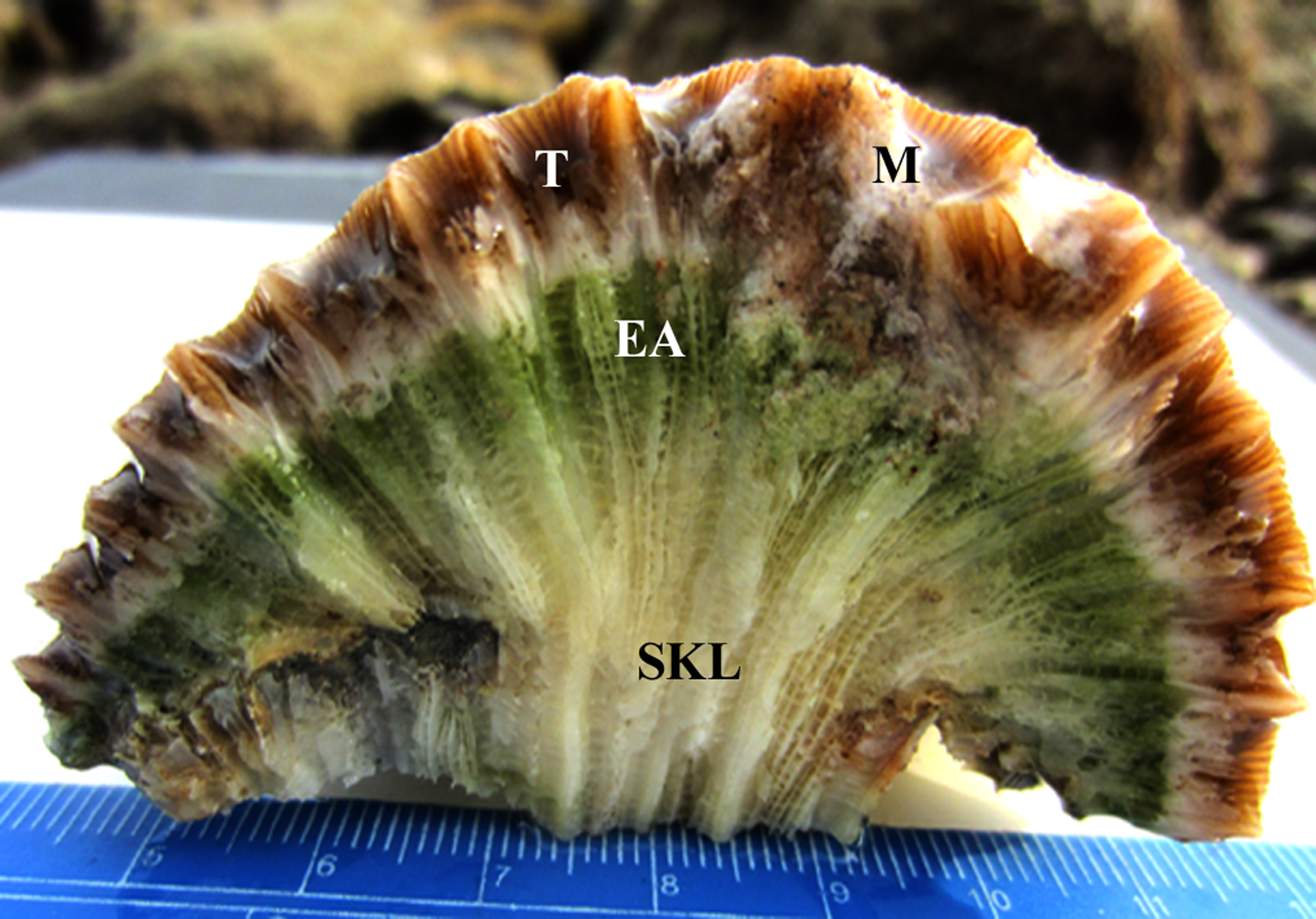

Supplement: S2 Fig — SKL = skeleton; EA = endolithic algae; M = mucus and T = tissue. (TIF) [file pone.0144902.s002.tif]
